# Aripiprazole for treatment of Attention-Deficit/Hyperactivity Disorder

**DOI:** 10.1192/j.eurpsy.2026.11078

**Published:** 2026-07-31

**Authors:** A. V. Samokhvalov

**Affiliations:** 1 Dr.Sam’s Health, Toronto; 2 Department of Psychiatry and Behavioural Neurosciences, McMaster University, Hamilton, Canada

## Abstract

**Introduction:**

Introduction: Attention-Deficit/Hyperactivity Disorder (ADHD) is a prevalent neurodevelopmental condition often associated with multiple comorbidities. While multiple effective treatment options for ADHD with proven efficacy exist, they might not be suitable in some cases primarily due to comorbid psychiatric and somatic conditions such as Bipolar Disorder (BD), Autism Spectrum Disorder (ASD) and others. In many of these cases the use of a partial agonist of dopaminergic receptors such as aripiprazole appears to be an optimal strategy both clinically and pathophysiologically.

**Objectives:**

To evaluate the effectiveness and feasibility of the use of aripiprazole for treatment of ADHD.

**Methods:**

Scoping review of literature.

**Results:**

PubMed literature search using specific keywords (“aripiprazole”, “ADHD”, “Attention-Deficit/Hyperactivity Disorder”) identified 175 entries, including large number of small observational studies and case reports involving primarily the use of aripiprazole for treatment of BD, ASD, and tics in patients with concurrent ADHD. The 2013 systematic review of the trials of aripiprazole for treatment of ADHD showed that aripiprazole was not an effective treatment option though it was limited to only 2 randomized controlled trials [1]. Most recently published reviews also produced mixed results indicating that second-generation antipsychotics, including aripiprazole can be helpful for treatment of specific symptoms and comorbidities with limited support for ADHD symptoms [2]. Overall, there is not enough evidence to support the use of aripiprazole for treatment of isolated ADHD, but there are indications that it may have some positive impact on ADHD symptoms when used for treatment of comorbidities such as BD.

**Conclusions:**

Data on the clinical effectiveness of aripiprazole for treatment of ADHD with and without comorbidities are scarce and do not support its use in isolated cases of ADHD, but it still may be of benefit in ADHD comorbid with other psychiatric conditions. Further research is warranted.

**Disclosure of Interest:**

None Declared

